# The Pathology of Morphine-Inhibited Nerve Repair and Morphine-Induced Nerve Damage Is Mediated via Endoplasmic Reticulum Stress

**DOI:** 10.3389/fnins.2021.618190

**Published:** 2021-02-19

**Authors:** Jie Liu, Shanyong Yi, Weibo Shi, Guozhong Zhang, Songjun Wang, Qian Qi, Bin Cong, Yingmin Li

**Affiliations:** ^1^Hebei Key Laboratory of Forensic Medicine, Collaborative Innovation Center of Forensic Medical Molecular Identification, College of Forensic Medicine, Hebei Medical University, Shijiazhuang, China; ^2^Research Center of Basic Medical Sciences, Department of Pathology, School of Basic Medical Sciences, Hubei University of Science and Technology, Xianning, China; ^3^School of Forensic Medicine, Xinxiang Medical University, Xinxiang, China

**Keywords:** morphine dependence, acute morphine exposure, cortex, ERS, Ki67, NURR1

## Abstract

**Objective:**

The aim of the present study was to observe the pathological damage in the cerebral cortex of rats under acute morphine exposure (AME) and different durations of morphine dependence (MD), explore whether endoplasmic reticulum stress (ERS) is involved in the damage process, and assess the effect of morphine exposure on the proliferation and differentiation of newborn neurons.

**Methods:**

Rat models of AME and different durations of MD were established. Pathological changes in cortical neurons were assessed by hematoxylin and eosin (H&E) and thionine staining. The expression of nuclear receptor-related factor 1 (NURR1) and that of the ERS-related proteins glucose-regulated protein 78 (GRP78), p-eIF2α, activating transcription factor 6 (ATF6), and CHOP in cortical neurons was assessed by immunohistochemistry. Double immunofluorescence labeling was used to observe the expression of Ki-67.

**Results:**

H&E and thionine staining revealed that AME resulted in pyknotic changes in cortical neurons. With prolonged morphine exposure, the number of pyknotic neurons was significantly increased, the protein expression of Ki-67 and NURR1 was significantly decreased, and the protein levels of GRP78, p-eIF2α, ATF6, and CHOP showed marked dynamic changes.

**Conclusion:**

AME and different durations of MD caused varying degrees of pathological changes in the cortex. Furthermore, the dynamic changes observed in ERS-related protein expression suggested that ERS may be associated with cortical injury. Different durations of MD inhibited the proliferation, differentiation, and migration of newborn neurons, which may affect the nerve repair process after injury.

## Introduction

A report by the World Health Organization on the abuse of morphine indicated that the misuse of this drug had increased in recent years (Volkow and Skolnick, 2012). Morphine dependence (MD) refers to a chronic recurrent brain disease characterized by the loss of self-control and compulsive, continuous drug seeking (Lamb and Ginsburg, 2018). Several studies have shown that MD can inflict varying degrees of injury on different systems of the body, including damage to the heart and lungs, direct effects on the central nervous system, and inhibitory effects on the respiratory center. Moreover, these effects may lead to acute or chronic cerebral ischemia and hypoxia, which, in turn, may cause nerve damage (Brailoiu et al., 2004; Luo et al., 2013; Rohbani et al., 2019). MD involves a complex series of pathophysiological changes in multiple brain regions, including, importantly, the cerebral cortex (Katebi et al., 2013; Adedayo et al., 2018); however, little is known about the pathomorphological changes induced by the misuse of this substance.

The endoplasmic reticulum (ER) is the primary organelle for protein synthesis, glycosylation, folding, secretion, and nascent protein transport. Under normal conditions, the protein folding ability of the ER matches the body’s protein synthesis requirements (Iurlaro and Muñoz-Pinedo, 2016; Kropski and Blackwell, 2018). However, when the body is stimulated by ischemia, hypoxia, injury, or other insults, the microenvironment of the ER changes, which can result in the accumulation of unfolded or misfolded proteins in the ER lumen and the subsequent induction of endoplasmic reticulum stress (ERS) (Xiang et al., 2017; Muneer and Shamsher Khan, 2019). Under ERS, the expression of glucose-regulated protein 78 (GRP78) increases, which can inhibit the synthesis of cellular proteins, accelerate the degradation of misfolded or unfolded proteins, and maintain ER homeostasis (Ibrahim et al., 2019). However, when ERS persists, protein kinase RNA (PKR)-like endoplasmic reticulum kinase (PERK), and activating transcription factor 6 (ATF6) can dissociate from GRP78, thereby activating downstream signaling pathways. Recent studies have shown that the activation of the ATF6 signaling pathway mainly exerts a cell-protective role (Hillary and FitzGerald, 2018), whereas the activation of the PERK/p-eIF2α signaling pathway can lead to the upregulation of CCAAT/-enhancer-binding protein homologous protein (CHOP) expression, and continuous CHOP expression can induce cell injury, or even cell death (Hu et al., 2019). Brain injury can induce the proliferation of endogenous neural stem cells (NSCs) and enable the newborn neurons to differentiate and migrate to the site of injury (Dai et al., 2019). Ki-67, a nuclear antigen, is often used as a marker for evaluating cell proliferation (Miller et al., 2018). Meanwhile, nuclear receptor-related factor 1 (NURR1), a member of the orphan nuclear receptor superfamily, is indispensable for neuronal differentiation, migration, maturation, and survival (Dong et al., 2016).

The activation status of ERS-related proteins and the proliferation and differentiation status of endogenous NSCs in acute morphine exposure (AME)- and chronic MD-induced cortical nerve damage remain unknown. In the present study, we first established rat models of AME as well as of different durations of MD. Subsequently, we investigated the pathological changes in cortical neurons, the alterations in the expression levels of ERS-related proteins, and changes in the proliferation, differentiation, and maturation status of newborn neurons aiming to provide pathomorphological evidence for the mechanisms underlying morphine-induced injury.

## Materials and Methods

### Animals

Adult male Wistar rats (Experimental Animal Center, Hebei Medical University, China), weighing 250 ± 20 g, were maintained in a room with an ambient temperature of 22 ± 2°C and a 12/12-h light/dark cycle, and had *ad libitum* access to food and water. This study was approved by the Institutional Review Board for Animal Experiments at the Hebei Medical University. Every attempt was made to reduce the number of animals used and to minimize animal pain and suffering. The rats were randomly divided into the following groups: 1-week control (Con), 3-week control, 6-week control, 1-week MD, 3-week MD, 6-week MD, 2-h control, and AME groups (*n* = 8 per group).

### Model of MD

As previously described (Shi et al., 2019), the model of MD was established through subcutaneous injections of morphine hydrochloride at increasing doses. Rats in the three morphine-dependence groups were subcutaneously injected in the back with morphine hydrochloride twice daily (08:00 and 20:00) for 5 days. The initial dose administered was 10 mg/kg and was increased by 10 mg/kg every other day until day 5 of treatment. The control rats received equal volumes of saline. The MD of model rats was confirmed after 5 days of morphine administration as described in Maldonado et al. (1992). Two rats were randomly selected from each control group and each morphine-dependent group and given a subcutaneous injection of naloxone hydrochloride (5 mg/kg) to induce withdrawal symptoms. Scoring involved observing signs of opiate withdrawal, including wet-dog shakes, stretching, cleaning fur, swallowing, standing, jumping, and teeth chattering. Following this assessment, 30 mg/kg morphine was administered twice daily (08:00 and 20:00) until 1-, 3-, or 6-weeks post-establishment of dependence.

Rats in the AME group were subcutaneously injected once in the back with morphine hydrochloride at a dose of 30 mg/kg. The control rats received an equal volume of saline.

### Tissue Preparation

Two hours after the last morphine or saline injection, the rats were deeply anesthetized and euthanized. The tissue used for staining was harvested and immediately fixed in 10% formalin, subsequently dehydrated using a graded ethanol series, and embedded in paraffin. Brain slices were obtained using a stereotaxic atlas (Paxinos and Watson, 2007) and a rotary microtome (Leica RM2255, Shanghai, China). Sections (5 mm) were prepared for thionine, immunohistochemical, and immunofluorescence staining and examined under a light microscope (Olympus IX71; Olympus, Tokyo, Japan).

### Reagents

Rabbit polyclonal antibodies against GRP78 (ab188878), CHOP (ab179823), and NURR1 (ab176184); the mouse monoclonal antibody against MAP2 (ab11268); and the rabbit monoclonal antibody targeting Ki-67 (ab16667) were purchased from Abcam (United States). Rabbit polyclonal antibodies against p-eIF2α (AF3087) and ATF6 (DF6009) were purchased from Affinity (China). The Alexa Fluor 488 donkey anti-mouse IgG (H + L) (1975519) and the Alexa Fluor 594 donkey anti-rabbit IgG (H + L) (1827674) secondary antibodies were purchased from Invitrogen (United States). The immunohistochemistry kit (SP9001) was purchased from the Zhongshan Goldenbridge Biotech, China, and the morphine hydrochloride for injection was produced in the First Pharmaceutical Factory of Shenyang, China.

### Body Weight Measurements

The body weight of the rats in all the groups was measured daily before treatment throughout the experiment.

### Hematoxylin and Eosin Staining

Deparaffinized sections were stained with hematoxylin for 2 min, transferred to 1% hydrochloric acid alcohol differentiation solution, and then stained with eosin for 3 s.

### Thionine Staining

Thionine staining was performed as previously described (Yi et al., 2019). Deparaffinized sections were stained with 4% thionine for 90 s at 60°C, dehydrated through a graded alcohol series, and mounted with neutral gum.

### Immunohistochemistry

Immunohistochemistry was performed as previously described (Yi et al., 2019). Antigen retrieval of deparaffinized sections was performed using a microwave, followed by incubation in 3% H_2_O_2_ in cold methanol for 30 min and blocking for 30 min using goat serum. The tissues were then incubated overnight at 4°C with antibodies against GRP78 (1:200), p-eIF2α (1:100), CHOP (1:200), ATF6 (1:100), and NURR1 (1:100). The next day, the tissues were incubated for 1 h with biotinylated secondary antibody and subsequently with horseradish peroxidase (HRP)-conjugated biotin for 30 min. Finally, DAB or AP-red was used as the chromagen. The tissues were counterstained with hematoxylin to demarcate locations in the sections. The primary antibodies were replaced with 0.01 mmol/L PBS in the negative controls (data not shown).

### Immunofluorescence Double Staining

Immunofluorescence was performed as previously described (Shi et al., 2019). The anti-Ki-67 antibody (1:100) was used as the first primary antibody and the anti-MAP2 antibody (1:150) as the second primary antibody. DyLight 594-conjugated AffiniPure goat anti-rabbit Ig (1:150) and DyLight 488-conjugated AffiniPure goat anti-mouse Ig (1:100) were used as the secondary antibodies.

### Cell Counting

Six rats from each group were used for morphological observation. One out of every three serial sections were selected for cell counting. Following a comparison of the sections after immunohistochemical staining and double immunofluorescence staining, the numbers of positive cells were counted in a field of view at 100× magnification. Two independent observers who were blinded to the experimental conditions performed the counts and calculated the average number of positive cells.

### Statistical Methods

The Kolmogorov–Smirnov test showed that the data were normally distributed in all groups (*P* > 0.1). The results are presented as mean ± SD. The data were analyzed by one-way ANOVA. Significance was defined as *P* < 0.05 for all statistical tests.

## Results

### Weight Change

**FIGURE 1:**
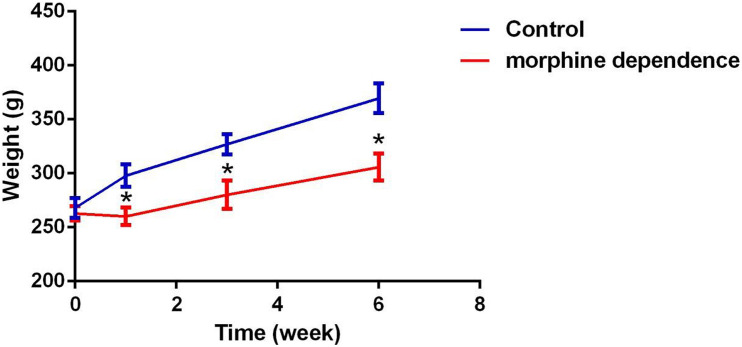
Effect of different durations of morphine dependence on the body weight. Compared with the control group, morphine dependence significantly reduced the body weight gain of rats. The results are shown as mean ± SD, *P < 0.05 vs. control group.

A change in body weight can be considered as a physiological indicator of morphine exposure. The weight of rats in the control group showed a marked increase after 1 week (297.78 ± 15.43), 3 weeks (327.08 ± 23.57), and 6 weeks (369.52 ± 28.81). In comparison, the weight of morphine-dependent rats was slightly decreased at 1 week (260.08 ± 8.18, *P* < 0.01), with slower increases being recorded at 3 weeks (280.18 ± 13.14, *P* < 0.05) and 6 weeks (305.64 ± 22.68, *P* < 0.05) after treatment (Figure 1).

### Hematoxylin and Eosin Staining Showed Pathological Changes in the Cerebral Cortex

**FIGURE 2:**
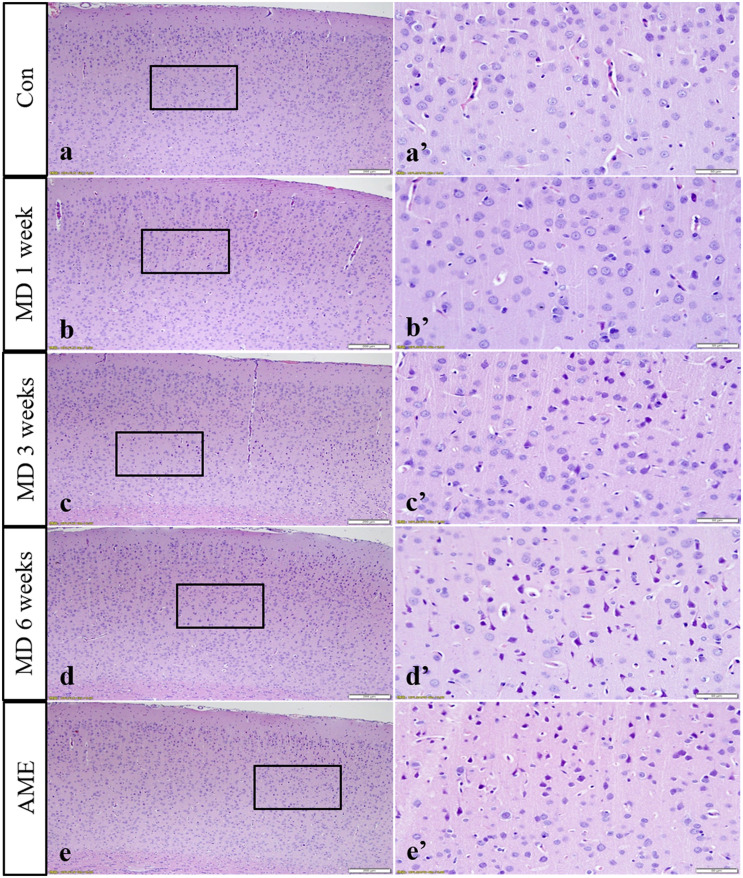
Hematoxylin staining of the cortex. Panels **(a’–e’)** are magnified areas of panels **(a–e)**, respectively. With increasing periods of morphine dependence, gaps around the small blood vessels and neurons widened, and microglia hyperplasia. Bars = 200 μm in panels **(a–e)**. Bars = 50 μm in panels **(a’–e’)**; MD, morphine dependence; AME, acute morphine exposure.

The cerebral cortices of rats in the control groups showed no pathological changes (data not shown), presenting a clear structure and neatly arranged neurons. Compared with the control group, no obvious change was observed after 1 week of MD. However, after 3 weeks of MD, the gaps around the small blood vessels and neurons widened, and microglial hyperplasia could be seen. Additionally, after 6 weeks of MD, tissue and cellular damage was more extensive and neurons were pyknotic and dying. Some pyknotic cells also appeared in the cortices of rats of the AME group (Figure 2).

### Thionine Staining Showed Pathological Changes in Cortical Neurons

**FIGURE 3:**
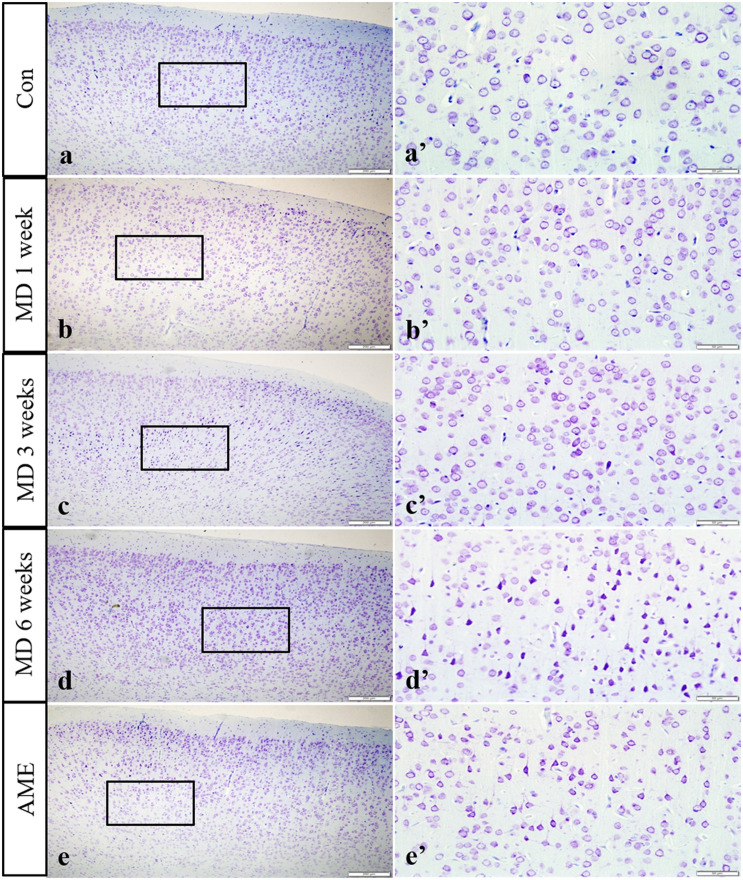
Thionine staining of the cortex. Panels **(a’–e’)** are magnified areas of panels **(a–e)**, respectively. With increasing periods of morphine dependence, Nissl body structures are not clear, and neurons are pyknotic and deeply stained. Bars = 200 μm in panels **(a–e)**. Bars = 50 μm in panels **(a’–e’)**; MD, morphine dependence; AME, acute morphine exposure.

In the control group, the neuronal structures were clear, and Nissl bodies were evenly distributed in the cytoplasm; a similar phenotype was observed after 1 week of MD. However, after 3 weeks of MD, a proportion of Nissl bodies had disappeared and pyknotic neurons were visible. Cellular damage was more extensive at 6 weeks. Meanwhile, some Nissl bodies had also disappeared in the AME group, and pyknotic neurons were also visible (Figure 3).

### GRP78, p-eIF2α, CHOP, ATF6, and NURR1 Expression in the Cerebral Cortex

Immunohistochemical staining showed that GRP78, p-eIF2α, ATF6, and CHOP proteins were located in the cytoplasm and were stained brown, while NURR1 protein was localized to the nucleus and was stained red.

**FIGURE 4:**
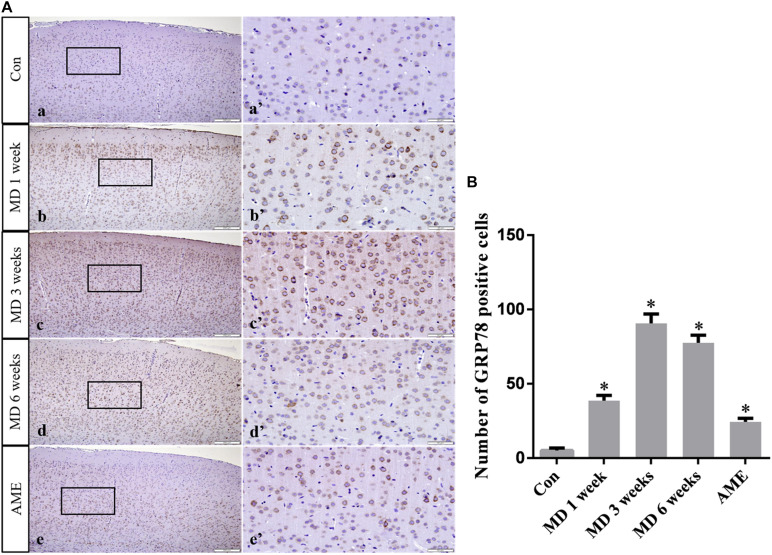
**(A)** Representative images showing GRP78 immunohistochemistry in the cortex. Panels **(a’–e’)** are magnified areas of panels **(a–e)**. Bars = 200 μm in panels **(a–e)**. Bars = 50 μm in panels **(a’–e’)**. **(B)** Quantitative analysis of the number of GRP78 positive cells. The data are shown as mean ± SD, *P < 0.05 vs. control group (*n* = 6). MD, morphine dependence; AME, acute morphine exposure.

ANOVA for GRP78-positive cells in the cerebral cortex showed that there were significant differences among the groups (*F* [4, 25] = 687.7; *P* < 0.0001). Compared with the control group (5.24 ± 1.55, *n* = 6), GRP78 expression was significantly upregulated in the 1-week MD (38.63 ± 3.51, *P* < 0.01, *n* = 6), 3-week MD (90.74 ± 6.25, *P* < 0.01, *n* = 6), 6-week MD (77.52 ± 5.15, *P* < 0.01, *n* = 6), and AME (24.4 ± 2.46, *P* < 0.01, *n* = 6) groups (Figure 4).

**FIGURE 5:**
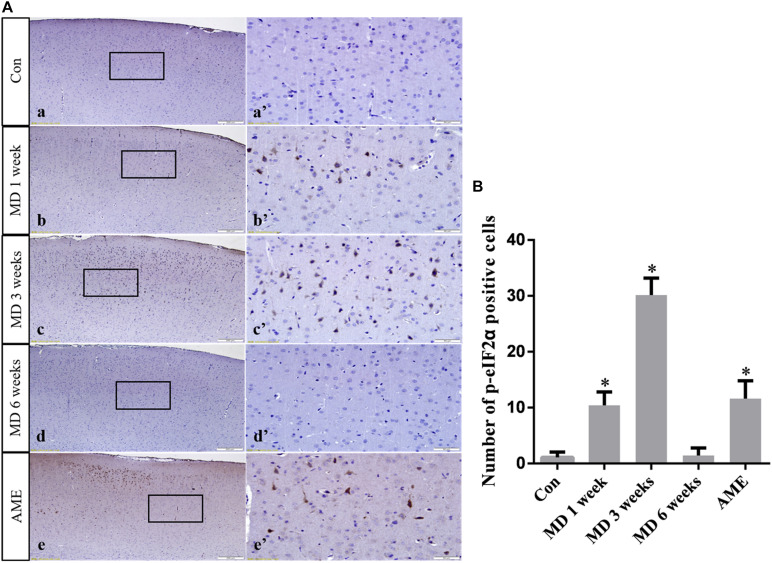
**(A)** Representative images showing p-eIF2α immunohistochemistry in the cortex. Panels **(a’–e’)** are magnified areas of panels **(a–e)**. Bars = 200 μm in panels **(a–e)**. Bars = 50 μm in panels **(a’–e’)**. **(B)** Quantitative analysis of the number of p-eIF2α positive cells. The data are shown as mean ± SD, ^∗^*P* < 0.05 vs. control group (*n* = 6). MD, morphine dependence; AME, acute morphine exposure.

ANOVA for p-eIF2α-positive cells in the cerebral cortex showed that there were significant differences among the groups (*F* [4, 25] = 99.58; *P* < 0.0001). Compared with the control group (1.12 ± 0.94, *n* = 6), p-eIF2α expression remained at a low level in the 6-week MD group (1.45 ± 1.34, *n* = 6), but was significantly upregulated in the 1-week MD (10.42 ± 2.41, *P* < 0.01, *n* = 6), 3-week MD (30.14 ± 3.03, *P* < 0.01, *n* = 6), and AME (11.62 ± 3.20, *P* < 0.01, *n* = 6) groups (Figure 5).

**FIGURE 6:**
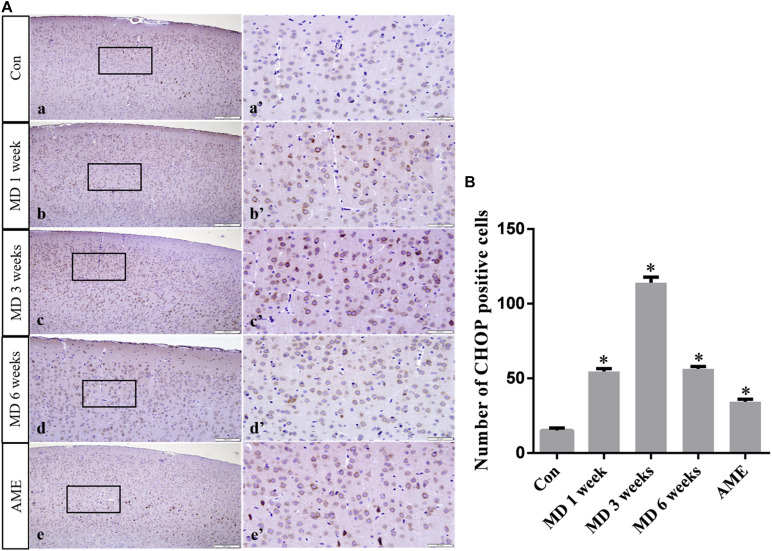
**(A)** Representative images showing CHOP immunohistochemistry in the cortex. Panels **(a’–e’)** are magnified areas of panels **(a–e)**. Bars = 200 μm in panels **(a–e)**. Bars = 50 μm panels in **(a’–e’)**. **(B)** Quantitative analysis of the number of CHOP positive cells. The data are shown as mean ± SD, ^∗^*P* < 0.05 vs. control group (*n* = 6). MD, morphine dependence; AME, acute morphine exposure.

ANOVA for CHOP-positive cells in the cerebral cortex showed that there were significant differences among the groups (*F* [4, 25] = 1493; *P* < 0.0001). Compared with the control group (15.23 ± 1.55, *n* = 6), CHOP expression was significantly upregulated in the 1-week MD (54.72 ± 1.89, *P* < 0.01, *n* = 6), 3-week MD (114.12 ± 3.67, *P* < 0.01, *n* = 6), 6-week MD (56.46 ± 1.43, *P* < 0.01, *n* = 6), and AME (34.46 ± 1.55, *P* < 0.01, *n* = 6) groups (Figure 6).

**FIGURE 7:**
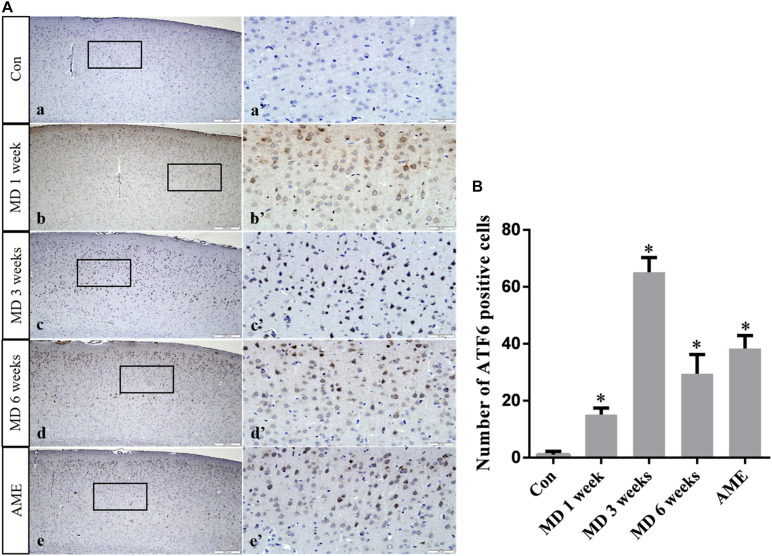
**(A)** Representative images showing ATF6 immunohistochemistry in the cortex. Panels **(a’–e’)** are magnified areas of panels **(a–e)**. Bars = 200 μm in panels **(a–e)**. Bars = 50 μm in panels **(a’–e’)**. **(B)** Quantitative analysis of the number of ATF6 positive cells. The data are shown as mean ± SD, ^∗^*P* < 0.05 vs. control group (*n* = 6). MD, morphine dependence; AME, acute morphine exposure.

ANOVA for ATF6-positive cells in the cerebral cortex showed that there were significant differences among the groups (*F* [4, 25] = 257.5; *P* < 0.0001). Compared with the control group (1.11 ± 1.14, *n* = 6), ATF6 expression was significantly upregulated in the 1-week MD (15.2 ± 2.22, *P* < 0.01, *n* = 6), 3-week MD (65.2 ± 5.07, *P* < 0.01, *n* = 6), 6-week MD (29.5 ± 6.74, *P* < 0.01, *n* = 6), and AME (38.34 ± 4.57, *P* < 0.01, *n* = 6) groups (Figure 7).

**FIGURE 8:**
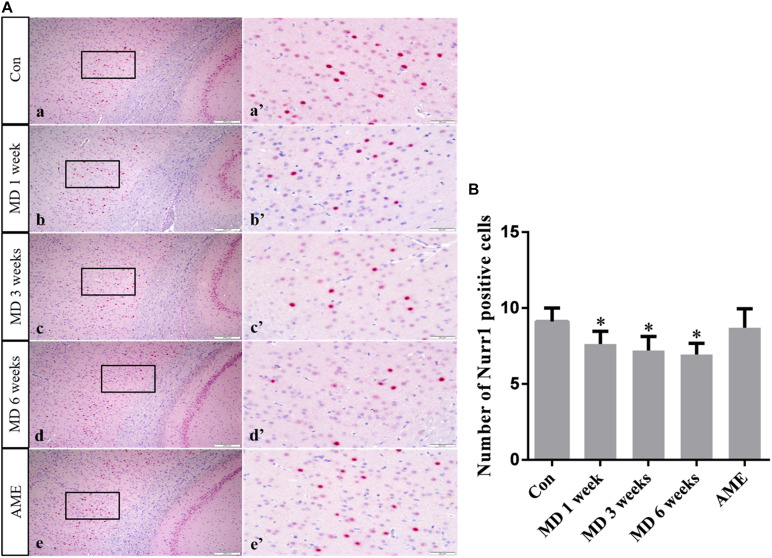
**(A)** Representative images showing Nurr1 immunohistochemistry in the cortex. Panels **(a’–e’)** are magnified areas of panels **(a–e)**. Bars = 200 μm in panels **(a–e)**. Bars = 50 μm in panels **(a’–e’)**. **(B)** Quantitative analysis of the number of Nurr1 positive cells. The data are shown as mean ± SD, ^∗^*P* < 0.05 vs. control group (*n* = 6). MD, morphine dependence; AME, acute morphine exposure.

ANOVA for NURR1-positive cells in the cerebral cortex showed that there were significant differences among the groups (*F* [4, 25] = 8.613; *P* = 0.0002). Compared with the control group (9.12 ± 0.88, *n* = 6), NURR1 expression remained at a high level in the AME group (8.71 ± 1.25, *n* = 6), but was significantly downregulated in the 1-week MD (7.63 ± 0.84, *P* < 0.01, *n* = 6), 3-week MD (7.21 ± 0.92, *P* < 0.01, *n* = 6), and 6-week MD (6.94 ± 0.74, *P* < 0.01, *n* = 6) groups (Figure 8).

### Ki-67 Expression Around the Ventricle

**FIGURE 9:**
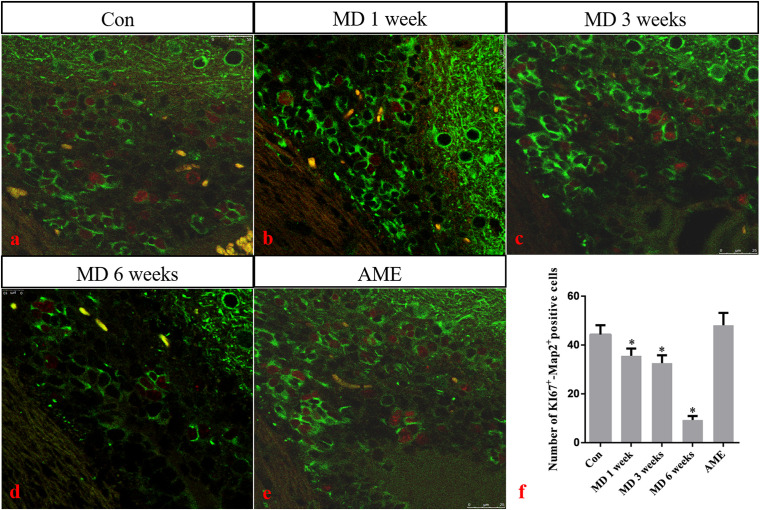
**(a–e)** Immunofluorescence staining for Ki67 (red) and Map2 (green) in the cortex. Bars = 25 μm in panels **(a–e)**. **(f)** Quantitative analysis of the number of Ki67+ – Map2+ positive cells. The data are shown as mean ± SD, *P < 0.05 vs. control group (*n* = 6). MD, morphine dependence; AME, acute morphine exposure.

Double-labeling showed that Ki-67 mostly co-localized with the neuronal marker MAP2 around the ventricle. Ki-67 protein was located in the nucleus and was stained red, and MAP2 protein was located in the cytoplasm and was stained green. ANOVA for Ki-67-positive cells in the cerebral cortex showed that there were significant differences among the groups (*F* [4, 25] = 163.4; *P* < 0.0001). Compared with the control group (44.32 ± 3.81, *n* = 6), the number of Ki-67/MAP2-positive cells remained at a high level in the AME group (48.12 ± 5.04, *n* = 6), and was significantly downregulated in the 1-week MD (35.62 ± 2.95, *P* < 0.01, *n* = 6), 3-week MD (32.63 ± 3.17, *P* < 0.01, *n* = 6), and 6-week MD (9.32 ± 1.63, *P* < 0.01, *n* = 6) groups (Figure 9).

## Discussion

Several studies have shown that long-term MD can cause acute or chronic cerebral ischemia and hypoxia by directly affecting the central nervous system and inhibiting the respiratory center, which can lead to central nervous system damage (Brailoiu et al., 2004; Volkow and Skolnick, 2012). Animal models allow for the identification and investigation of mechanisms underlying the effects of MD. In the present study, we established rat models of AME and different durations of MD aiming to better understand the mechanisms involved in morphine exposure-induced injury on the body and provide treatment and prevention strategies.

The cerebral cortex comprises a layer of neurons and synapses located on the surface of the cerebral hemispheres. The cerebral cortex is folded into gyri, and approximately two-thirds of it is submerged inside brain fissures. The cortex is involved in higher mental functions, general movements, functions of the viscera, perception, and behavioral reactions (Cadwell et al., 2019). However, the changes induced in cortical neurons by MD or AME remain poorly understood. In the present study, the results of hematoxylin and eosin (H&E) and thionine staining revealed that long-term MD and AME resulted in pyknotic neurons and a reduction in the number of Nissl bodies, which suggested that long-term MD and AME can cause nerve damage, and that, with increasing periods of morphine exposure, nerve damage can become more extensive.

There is an important link between ERS and body damage. Several studies have demonstrated that ERS not only participates in the occurrence and progression of a variety of neurodegenerative diseases, but also plays a critical role in the pathology of nerve cell dysfunction (Johnson et al., 2011; Xiang et al., 2017; Muneer and Shamsher Khan, 2019). Under ERS, the protective mechanisms of the cell are activated, namely, the unfolded protein response (UPR), which results in the upregulation of the expression of GRP78, a molecular chaperone. GRP78 can bind to misfolded or unfolded proteins, thereby reducing the burden of the ER and restoring its function (Ni et al., 2011). In the present study, the expression level of GRP78 in the AME group was significantly higher than that in the control group, and, with increasing periods of morphine exposure, the expression level of GRP78 showed a significantly increasing trend. This suggested that ERS occurred in the cortex of morphine-dependent rats, and that the body’s protective mechanisms were activated.

When morphine exposure persists, signaling pathways downstream of ERS will be activated, which can result in cell damage, or even cell death (Omura et al., 2013; Zhang et al., 2017). This may explain the shrinkage, disappearance of Nissl bodies, and other increasingly serious pathological impairments observed in this study.

Activating transcription factor 6 is a type-II transmembrane glycoprotein that, under ERS, can be transferred from the ER to the Golgi apparatus, where it is cleaved by site-1 and site-2 proteases. The N-terminal fragment, containing the alkaline leucine zipper domain, translocates into the nucleus and acts as a transcription factor that can activate the expression of ERS-related genes by combining ERS response elements and UPR elements (Papaioannou et al., 2018). Because the ERS response triggered by the selective activation of ATF6 is conducive to virus replication and the maintenance of cell viability, it is currently believed that the primary role of the ATF6 signaling pathway is the promotion of cell survival (Hillary and FitzGerald, 2018). In the present study, the expression level of ATF6 in the AME group was significantly higher than that in the control group, and, with increasing periods of morphine exposure, the expression level of ATF6 increased in the initial stages of MD, and then decreased at 6 weeks. These results showed that the protective effect of ATF6 on cortical neurons was significantly weakened with prolonged morphine exposure, leading to the activation of a series of downstream signaling pathways that induced cell injury and/or cell death.

CHOP, also known as growth arrest and DNA-damage inducible gene 153 (GADD153), is a key, ERS-specific proapoptotic transcription factor. Under normal physiological conditions, CHOP is expressed at a very low level (Yi et al., 2019). The PERK/eIF2α signaling pathway plays a dominant role in inducing CHOP expression (Rozpedek et al., 2016). PERK can inhibit the effect of eIF2B by promoting the phosphorylation of eIF2α, which can reduce protein translation and the amount of unfolded protein, thereby maintaining cell survival. When ERS is severe or prolonged, upstream open reading frame regulatory sequences present in the 5’-untranslated regions of mRNAs become activated, which inhibits eIF2α-dependent protein translation and activates the downstream ATF4/CHOP signaling pathway. Continuous expression of CHOP can cause cell damage, or even cell death (Cubillos-Ruiz et al., 2017; Wu et al., 2018; Lorenzon-Ojea et al., 2020). In this study, the expression levels of p-eIF2α and CHOP in the AME group were significantly higher than those of the control group. With increasing periods of morphine exposure, the expression level of p-eIF2α first increased, and then underwent a significant decline at 6 weeks, while CHOP showed sustained high levels of expression. These results suggested that the protective and damaging effects of ERS on neurons were in a state of mutual restraint in the 1- and 3-week MD groups. Subsequently, the high level of CHOP expression could lead to cell injury and death, which was consistent with our observations of pathological changes in cortical neurons after 6 weeks of MD. These data indicated that the PERK/p-eIF2α/CHOP pathway is associated with morphine exposure-induced cortical neuronal injury.

Ki-67 is a nuclear antigen specifically expressed by proliferating cells and is commonly used to evaluate tumor proliferative ability, malignancy, and prognosis (Scholzen and Gerdes, 2000; Miller et al., 2018). Under normal circumstances, Ki-67 is expressed in multiple brain regions, especially around the ventricle. Given that Ki-67-positive cells around the ventricle are NSCs and neural progenitor cells (NPCs), the expression of Ki-67 likely reflects the proliferation of nerve cells. In the present study, double immunofluorescence labeling of the neuron-specific marker MAP2 and Ki-67 were used as indicators of the proliferative status of NSCs and NPCs around the lateral ventricle after morphine exposure. No significant differences were observed between the AME and control groups, while the number of double-labeled Ki-67/MAP2-positive cells around the lateral ventricle showed a decreasing trend with increasing periods of morphine exposure. These results suggested that MD suppressed the proliferation of NSCs and NPCs around the lateral ventricle. We have previously shown that NURR1 plays an important role in the differentiation, migration, and maturation of atypical dopaminergic neurons outside the midbrain (Li et al., 2011, 2012). Although NURR1 is mainly expressed in dopaminergic neurons in the ventral side of the midbrain, it is also expressed in layers II–V of the cortex (Sano et al., 2008). In the present study, NURRl expression was significantly decreased in the morphine-dependent groups. These results suggested that MD inhibited the differentiation, migration, and maturation of cortical neurons, which may result in a decrease in the number of newborn neurons.

In conclusion, the results of this study demonstrated that ERS is involved in morphine exposure-induced injury. Moreover, morphine exposure inhibited the proliferation of endogenous NSCs and the differentiation and maturation of newborn neurons, thereby affecting nerve repair. We believe these findings provide morphological evidence for the mechanisms involved in morphine-induced cortical neuron injury and the repair process after brain injury.

## Data Availability Statement

The original contributions presented in the study are included in the article/supplementary material, further inquiries can be directed to the corresponding author/s.

## Ethics Statement

The animal study was reviewed and approved by the Institutional Review Board for Animal Experiments at the Hebei Medical University.

## Author Contributions

JL and SY designed and performed the experiments. WS and GZ performed the statistical analysis and organized the data. SW and QQ created the figures. BC and YL supervised the research design and revised the manuscript. All the authors read and approved the final version of the manuscript.

## Conflict of Interest

The authors declare that the research was conducted in the absence of any commercial or financial relationships that could be construed as a potential conflict of interest.
